# Mini-Multilocus Sequence Typing Scheme for the Global Population of *Neisseria gonorrhoeae*

**DOI:** 10.3390/ijms25115781

**Published:** 2024-05-26

**Authors:** Ilya Kandinov, Boris Shaskolskiy, Dmitry Kravtsov, Marina Filippova, Anatoliy Larkin, Dmitry Gryadunov

**Affiliations:** Center for Precision Genome Editing and Genetic Technologies for Biomedicine, Engelhardt Institute of Molecular Biology, Russian Academy of Sciences, 119991 Moscow, Russia; b.shaskolskiy@gmail.com (B.S.); solo13.37@yandex.ru (D.K.); mafilippova@mail.ru (M.F.); larkinanatanat@yandex.ru (A.L.); grad@biochip.ru (D.G.)

**Keywords:** *Neisseria gonorrhoeae*, multilocus sequence typing, genogroups, phylogenetic analysis, informative polymorphisms, antimicrobial resistance, oligonucleotide microarray

## Abstract

The increasing problem of antimicrobial resistance in *N. gonorrhoeae* necessitates the development of molecular typing schemes that are suitable for rapid and mass screening. The objective of this study was to design and validate a mini-MLST scheme for *N. gonorrhoeae* based on global pathogen population data. Using sequences of seven housekeeping genes of 21,402 isolates with known MLSTs from the PubMLST database, we identified eighteen informative polymorphisms and obtained mini-MLST nucleotide profiles to predict MLSTs of isolates. We proposed a new MLST grouping system for *N. gonorrhoeae* based on mini-MLST profiles. Phylogenetic analysis revealed that MLST genogroups are a stable characteristic of the *N. gonorrhoeae* global population. The proposed grouping system has been shown to bring together isolates with similar antimicrobial susceptibility, as demonstrated by the characteristics of major genogroups. Established MLST prediction algorithms based on nucleotide profiles are now publicly available. The mini-MLST scheme was evaluated using a MLST detection/prediction method based on the original hydrogel DNA microarray. The results confirmed a high predictive ability up to the MLST genogroup. The proposed holistic approach to gonococcal population analysis can be used for the continuous surveillance of known and emerging resistant *N. gonorrhoeae* isolates.

## 1. Introduction

Gonorrhea is a sexually transmitted infection considered by the World Health Organization to be a serious threat to the reproductive health of the population [1]. Clinical isolates of *N. gonorrhoeae* resistant to third-generation cephalosporins and macrolides have already been found in the global population, suggesting that the therapeutic potential of these antibiotics may soon be exhausted [2,3].

Molecular typing is an essential tool for monitoring gonococcal infection. It aims to identify the most pandemically important genotypes (sequence types, ST) and estimate transmission networks [4,5]. Due to the high genetic variability of *N. gonorrhoeae*, new sequence types emerge in the population, which requires time and resources for analysis. The diversity of STs also poses a challenge for a comprehensive epidemiological analysis. Population genetic studies frequently group individual sequence types into genogroups. This simplifies the analysis of phylogenetically close isolates of different sequence types, making it easier to determine the possible causes of the emergence of drug-resistant strains in the population [6,7,8,9].

Multilocus Sequence Typing (MLST) is a widely used typing method for various bacterial species that allows efficient association of isolates with different genetic lineages [10]. MLST uses highly conserved, slow-evolving genes, and their sequences are available for most bacteria, including *Neisseria* spp., in the PubMLST database (https://pubmlst.org/). The MLST typing system was initially proposed by Maiden et al. to standardize the monitoring of clonal lines of *N. meningitidis*, the closest related organism of *N. gonorrhoeae*, between laboratories [11]. Genotyping of *N. gonorrhoeae* according to the MLST scheme is based on the determination of nucleotide sequences of seven loci of housekeeping genes: *abcZ* (ABC transporter), *adk* (adenylate kinase), *aroE* (shikimate dehydrogenase), *fumC* (fumarate hydratase), *gdh* (glucose-6-phosphate dehydrogenase), *pdhC* (pyruvate dehydrogenase subunit), and *pgm* (phosphoglucomutase). Based on the sequencing results, a unique allele number is obtained for each locus from the database, and new alleles are numbered chronologically according of their addition to the database [12]. A molecular sequence type number is assigned to the analyzed isolate based on the set of identified alleles. As of 2023, the PubMLST database (accessed on 15 December 2023) contains 1442 molecular sequence types for *N. gonorrhoeae*. The MLST typing scheme is employed for the analysis of gonococcal populations with a view to epidemiological surveillance [13].

*N. gonorrhoeae* isolates belonging to certain MLST types possess “dangerous” allelic variants of antibiotic resistance genes, including the penicillin-binding protein 2 (PBP2) gene (*penA*) [14] or the mtrCDE efflux pump operon [15,16]. For example, the *penA* gene allele “XXXIV” and “X” mosaic types are common in isolates ST1901 and ST7363, which are widely distributed in the global population of *N. gonorrhoeae* and associated with decreased susceptibility to third-generation cephalosporins due to the *penA* gene allele [14]. These and similar mosaic alleles have probably evolved as a result of horizontal transfer from other microorganisms of the *Neisseria spp* [14,17]. Such *penA* gene variants encode PBP2 with decreased affinity to β-lactams, which reduces the susceptibility of the pathogen to third-generation cephalosporins [18]. These isolates were first reported in Japan and the European Union, and later spread globally due to transboundary transmission and selective pressure from antimicrobial agents [19,20]. ST9363 is another significant sequence type present in the global population of *N. gonorrhoeae*. Isolates of this ST are associated with azithromycin resistance and possess mosaic variants of genes that encode the MtrCDE and its repressor protein MtrR [16]. The global dissemination of these genetic lines, combined with reduced susceptibility or resistance to recommended antimicrobials, may result in the ineffectiveness of current treatment protocols. These facts highlight the significance of utilizing and optimizing current genotyping methods for *N. gonorrhoeae*. These methods are crucial for molecular epidemiological surveillance and allow the prompt detection of hazardous clones.

Genotyping of *N. gonorrhoeae* isolates according to the MLST scheme is traditionally performed by Sanger sequencing of *abcZ*, *adk*, *aroE*, *fumC*, *gdh*, *pdhC*, and *pgm* gene fragments [21]. The procedure involves separate amplification of each fragment followed by purification of PCR products, sequencing reactions, and determination of the sequence of a single locus using a capillary sequencer, which is quite labor-intensive. High-throughput sequencing (NGS) technologies are used for genotyping *N. gonorrhoeae* isolates.

The cgMLST (core genome Multilocus Sequence Typing) involves the evaluation of genes that identify the origin of a clinical isolate and the genetic determinants of antibiotic resistance [22]. The cgMLST uses 1659 genes of both chromosomal and plasmid localization. Although informative, this method has inherent drawbacks such as complex sample preparation and expensive reagent kits [4]. As a result, cgMLST is not yet a universal replacement for routine MLST and NG-MAST (*Neisseria gonorrhoeae* multi-antigen sequence typing).

An alternative and less labor-intensive method of genotyping is the MLST-based scheme, which predicts genotypes by analyzing informative single-nucleotide polymorphisms (mini-MLST) [23,24]. Due to the high conservatism of the genes used for MLST, polymorphisms are typically found in specific positions. Analyzing these positions can accurately predict the MLST genotype, eliminating the need to sequence all seven loci. Genotyping isolates using the mini-MLST scheme significantly reduces the time and labor required for a single study and is suitable for rapid and mass screening [25].

Whiley et al. demonstrated the feasibility of predicting the MLST genotype of *N. gonorrhoeae* using a small number of informative single-nucleotide polymorphisms (SNPs) in the corresponding genes [26]. Using real-time PCR and high-resolution melting curve analysis, 14 informative polymorphisms in *abcZ*, *adk*, *aroE*, *fumC*, *gdh*, *pdhC*, and *pgm* genes were identified [26]. After completing the amplification process, a profile of informative SNPs was obtained for the isolates. This profile was then compared to known profiles from the database to predict the MLST genotype of the isolate. The method was assessed using 86 isolates of *N. gonorrhoeae* [26]. Later, on a sample of 397 isolates, it was demonstrated that the MassARRAY iPLEX platform can identify single-nucleotide polymorphisms and predict *N. gonorrhoeae* MLST genotype [27]. The mini-MLST scheme thus appears to offer a promising approach for the typing of *N. gonorrhoeae*. Nevertheless, these studies were conducted on a limited number of *N. gonorrhoeae* isolates and did not reflect the associations between mini-MLST genotypes and resistance to antimicrobials.

The study aims to develop and validate a mini-MLST scheme for *N. gonorrhoeae* using global population data, including target gene sequences and antimicrobial susceptibility of isolates. The objectives of the study were to generate mini-MLST profiles that correspond to MLST genotypes, to characterize the MLST genogroups formed from the derived MLST genotypes, establish their phylogenetic relatedness, and examine their associations with antimicrobial resistance. The mini-MLST scheme for *N. gonorrhoeae* was performed using a hydrogel DNA microarray-based MLST genotype detection/prediction method.

## 2. Results

### 2.1. Identification of Informative Polymorphisms in the MLST-Related Genes of N. gonorrhoeae and Construction of Mini-MLST Profiles

**Figure 1:**
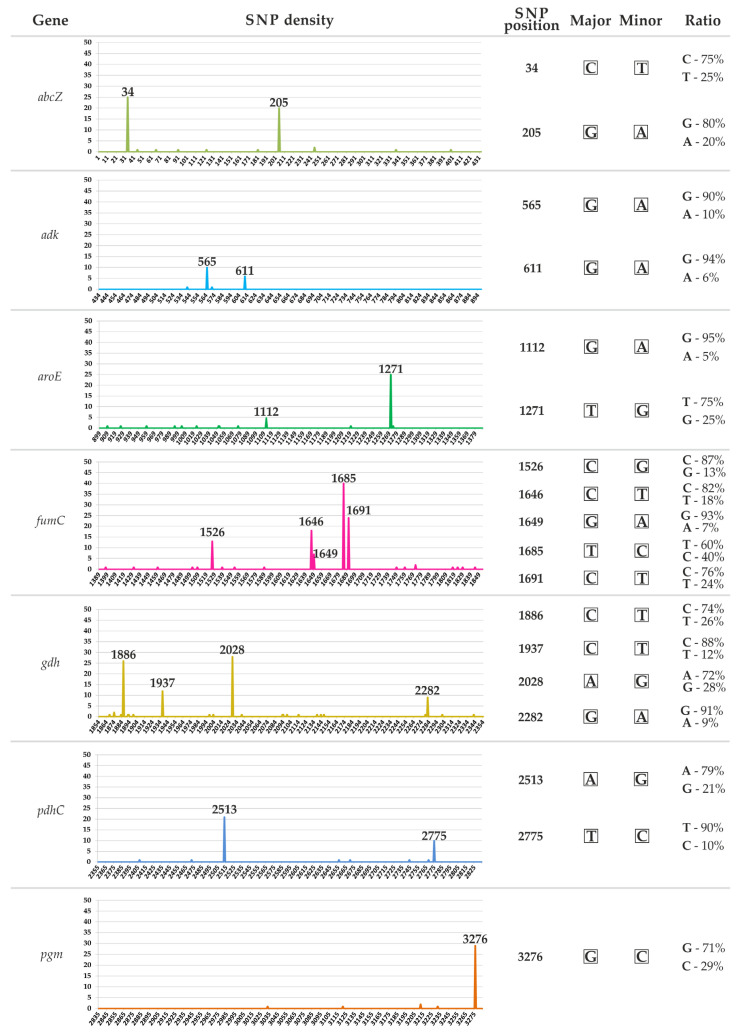
SNP density distribution at seven gene loci used for *N. gonorrhoeae* MLST. The *Y*-axis represents the height of peaks, which corresponds to the frequency of nucleotide substitutions (%) at each of the 3286 positions on the *X*-axis. This indicates the proportion of minor nucleotides relative to major nucleotides at each position. SNP density of 0% indicates the presence of only the major nucleotide at a particular position, while a density of 50% indicates an equal ratio of major and minor nucleotides at that position. The graphs on the right show the positions of nucleotide substitutions and the ratio of major to minor nucleotides in those positions. The colors correspond to the different loci analyzed.

The SNP density values for the seven housekeeping gene loci were obtained based on sequence analysis of 21,402 isolates with known MLSTs from the PubMLST database (Figure 1). We identified 18 significant positions where informative polymorphisms are most frequently found in the multiple alignment. These positions were: 34 and 205 in the *abcZ* gene, 565 and 611 in *adk*, 1112 and 1271 in *aroE*, 1526, 1646, 1649, 1685, and 1691 in *fumC*, and 1886, 1937, 2028, and 2282 in *gdh*. Additionally, positions 2513 and 2775 in *pdhC* and position 3276 in *pgm* also showed significant polymorphisms. A total of 1442 mini-MLST profiles were obtained for all sequencing types of *N. gonorrhoeae* in the global population (Table S1, Supplementary Materials).

### 2.2. MLST Genogroups as a Stable Characteristic for the Global Population of N. gonorrhoeae

In total, 101 genogroups with 687 MLSTs were identified based on the results of mini-MLST profile analysis of 1442 sequencing types found in the global population of *N. gonorrhoeae*. The remaining sequence types were categorized as ‘double’ and ‘single’ strains, with 242 and 513 MLSTs, respectively. Therefore, a new system for grouping *N. gonorrhoeae* MLSTs based on mini-MLST profiles has been proposed.

**Figure 2:**
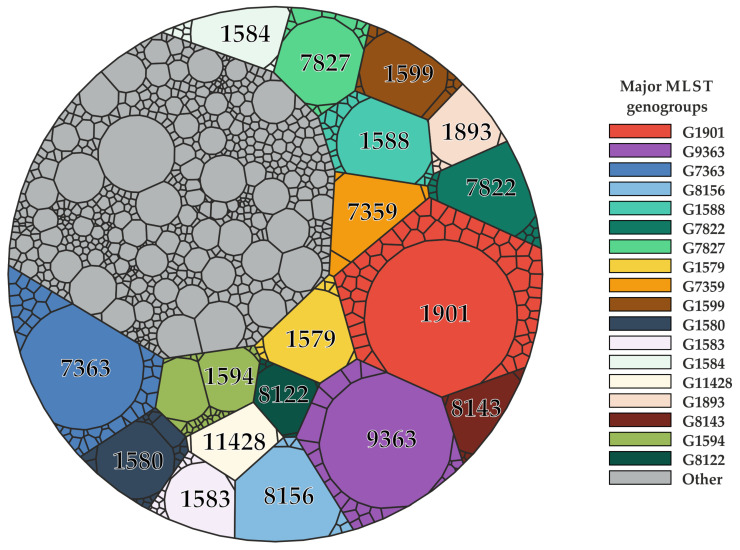
Voronoi diagram for the obtained MLST genogroups of *N. gonorrhoeae*. Each cell represents an MLST, and its size corresponds to the frequency of the presence of each sequence type in the global population of *N. gonorrhoeae* (%). Colors in the diagram indicate major genogroups. Other genogroups, as well as ‘double’ and ‘single’ strains, are highlighted in gray. The colors representing the genogroups in Figure 2, Figure 3 and Figure 4 are the same.

The frequency of each sequence type was calculated for the world population, as well as for the genogroup (Table S1, Supplementary Materials). Out of the 101 MLST genogroups, the *N. gonorrhoeae* global population was most commonly affected by 18 major genogroups, including G1901, G9363, G7363, etc. A Voronoi diagram (Figure 2) was created for the MLST genogroups obtained, as well as for the STs assigned to both ‘double’ and ‘single’ strains.

**Figure 3:**
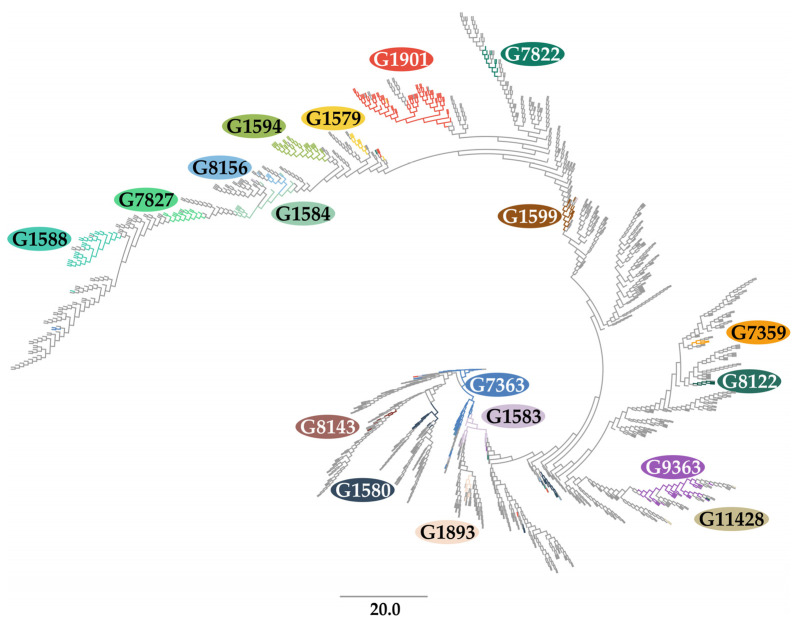
Circular phylogram of MLST genogroups. The tree has a total of 1442 terminal nodes, each representing an MLST. Major genogroups are indicated by colors in the phylogram, while other genogroups, as well as ‘double’ and ‘single’ strains, are highlighted in gray. The colors used to represent the genogroups in Figure 2, Figure 3 and Figure 4 are the same.

The diagram illustrates that typically, one sequence type dominates the majority of each genogroup, accounting for an average of 91%. The proportion of other sequence types within a genogroup is negligible. For instance, ST1901 makes up 94% of the G1901 genogroup, with only a small portion being occupied by other sequence types like ST6814, ST9365, ST9751, etc. The higher MLST sequence number of these STs suggests that they were discovered in the global population of *N. gonorrhoeae* at a later time and likely originated from a common ancestor. To analyze the evolutionary relationships between different MLSTs and confirm the accuracy of our proposed system for combining sequence types into genogroups, we conducted a phylogenetic analysis. The results are shown in Figure 3.

The phylogram indicates that sequence types within an MLST genogroup are closely related phylogenetically, and each genogroup usually dominates the corresponding clade on the tree. Table S1A provides information on the clade analyses, including each of the mini-MLST profiles detected. It also shows the proportions of leaves that make the main clade polyphyletic, as well as the proportions of isolates corresponding to these leaves and the leaves of isolates from the main clade. The ability to accurately predict the MLST type is 90%. Even when the MLST type is not accurately determined, the predicted type is phylogenetically similar in more than 98% to the isolate with the dominant MLST. For instance, in the G1901 genogroup, which has the largest number (59) of sequence types, only 6% of isolates belong to another MLST; most of the G1901 genogroup isolates (98%) belong to the one clade. Therefore, the MLST phylogeny confirms the accuracy of our proposed genogrouping system and allows us to accurately distinguish between distantly related sequence types using the 18 informative polymorphisms.

It is important to note that in the sample, there were sequence types with a large number of substitutions in the loci under study and were phylogenetically distant from all other sequence types. These STs accounted for approximately 0.5% of the total. The sequences of such STs were similar to those of *N. meningitidis*, for example, MLST: 12529, 12239, 13961, 15271, 15272, 15616, and 16263. These genetic anomalies in the analyzed conserved genes of *N. gonorrhoeae* may have resulted from horizontal gene transfer or errors in bacterial species identification during culture analysis.

### 2.3. Association of MLST Genogroups with Antimicrobial Susceptibility of N. gonorrhoeae Isolates

**Figure 4:**
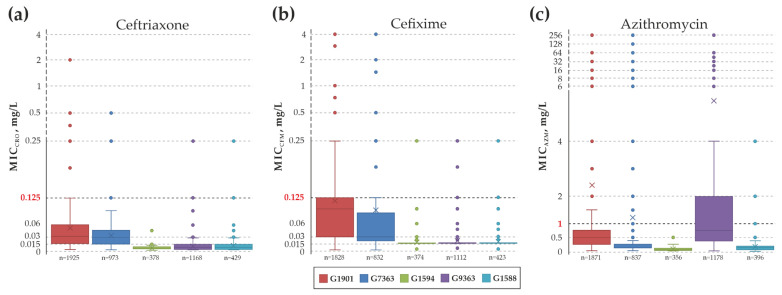
Box plots: MIC of ceftriaxone (**a**), cefixime (**b**), azithromycin (**c**) in isolates belonging to major MLST genogroups. The median MIC values of the antibiotics for each group are represented by the thick solid lines in each box. The mean MIC value for each group is indicated by the ‘×’ symbol. The interquartile range is shown by the whiskers. The thick dashed line and the red value indicate the MIC threshold that separates susceptible and resistant isolates (EUCAST criteria). The scales of large MIC values have equal distances and are indicated by a dashed line. The genogroups in Figure 2, Figure 3 and Figure 4 are represented by the same colors.

We analyzed the susceptibility of *N. gonorrhoeae* isolates to ceftriaxone, cefixime, azithromycin in the formed MLST genogroups. Box plot diagrams in Figure 4 illustrate the difference in MIC values of antimicrobial drugs in isolates from major MLST genogroups G1901, G7363, G1594, G9363, and G1588. The results of the MIC comparisons between the above genogroups are shown in Supplementary Table S4. A statistically significant difference (*p*-value << 0.001) in MIC value for ceftriaxone, cefixime, azithromycin was found for all genogroups, depicted in the Figure 4 (1588, 1594, 1901, 7363, 9363).

Figure 4a demonstrates that isolates from genogroup G1901 are mostly associated with reduced susceptibility to ceftriaxone. The whiskers maximum value reaches 0.125 mg/L, which corresponds to the threshold value separating susceptible and resistant isolates according to EUCAST criteria. The highest MIC_CRO_ emissions are observed in G1901 genogroup, reaching up to 2 mg/L. Similar emissions are also observed in G7363, G9363, and G1588 genogroups. However, their upper quartiles and maximum whisker values are below the resistance threshold of 0.125 mg/L. The lowest interquartile range value and outliers are observed in isolates G1594, which were all found to be susceptible to ceftriaxone. Figure 4b shows a similar distribution of MICs by groups for cefixime. The upper quartile of G1901 genogroup is at 0.125 mg/L, and the maximum whisker value reaches 0.25 mg/L, which is already above the EUCAST threshold. The maximum value of whiskers in G7363 genogroup reaches 0.125 mg/L, but the upper quartile is below this threshold. Isolates of G1594, G9363 and G1588 genogroups are generally susceptible to cefixime except for rare outliers up to 0.25 mg/L.

The EUCAST (ECOFF) resistance threshold for azithromycin is 1 mg/L. Figure 4c indicates that G9363 genogroup isolates are most commonly linked to azithromycin resistance. The upper quartile of G9363 is located at 2 mg/L, and the maximum whisker value is located at 4 mg/L. Outliers up to 256 mg/L are found in G9363, G1901, and G7363, which are likely associated with spontaneous mutations in 23S rRNA genes [28,29]. Similarly to ceftriaxone and cefixime, all isolates of G1594 genogroup were susceptible to azithromycin and also had the lowest interquartile range value.

### 2.4. Mini-MLST Software Tool for N. gonorrhoeae

We have created and launched an online tool, which is publicly available at (https://minimlst.su/, accessed on 15 December 2023), for analyzing *N. gonorrhoeae* isolates. Additionally, online access is available for the 1442 mini-MLST nucleotide profiles for all MLSTs currently described in the global *N. gonorrhoeae* population (refer to Table S1 in the Supplementary Materials). The algorithm predicts the MLST of an isolate using data from 18 informative polymorphisms through nucleotide profile mini-MLST (https://minimlst.su/#snptomlst, accessed on 15 December 2023). It obtains the nucleotide profile from MLST (https://minimlst.su/#mlsttosnp, accessed on 15 December 2023), and allows for the reverse procedure to obtain the nucleotide profile of mini-MLST associated with a given ST.

Note that the typing scheme provides an accurate prediction up to the MLST genogroup, since all STs within the same genogroup have a completely identical mini-MLST profile. The algorithm at https://minimlst.su/#gmlst, accessed on 15 December 2023 can identify the MLST genogroup of the selected sequence type. If the entered nucleotide profile is associated with only one profile, which means it belongs to a single strain, the sequencing type of the isolate can be determined with 100% accuracy. The algorithms mentioned above are also available for download on GitHub (https://github.com/AnatoliyLarkin/mini-MLST, accessed on 1 April 2024) and in Supplementary File S2.

### 2.5. Genotyping N. gonorrhoeae Isolates Using a Mini-MLST Microarray-Based Assay

**Figure 5:**
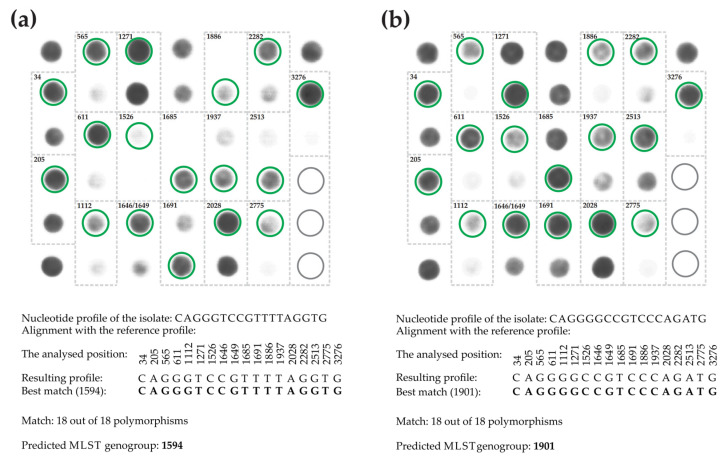
Fluorescence hybridization patterns of the microarray after the analysis of genomic DNA from *N. gonorrhoeae* isolates. Dashed lines highlight sets of elements for each analyzed position. Green circles indicate elements with maximum signals corresponding to perfect hybridization duplexes for certain SNPs. The mini-MLST profile consists of 18 informative SNPs, listed below, which are compared to reference sequence type-specific profiles from Table S1 (Supplementary Material) using the best-match principle to predict the MLST genogroup. (**a**) The analysis of the *N. gonorrhoeae* MLST 1594 isolate resulted in its assignment to the MLST G1594 genogroup, based on the mini-MLST profile obtained (CAGGGTCCGTTTTAGGTG). (**b**) The analysis of the *N. gonorrhoeae* MLST 1901 isolate resulted in its assignment to the MLST G1901 genogroup based on the obtained mini-MLST profile (CAGGGGCCGTCCCAGATG).

Hybridization analysis on hydrogel microarrays was used to identify informative polymorphisms, obtain mini-MLST profiles, and analyze 107 clinical isolates of *N. gonorrhoeae* from Russia. These isolates had previously been analyzed using Sanger sequencing for MLST [30]. Figure 5 shows examples of microarray fluorescent images after analysis, displaying hybridization patterns.

Table S3 (Supplementary Material) summarizes the results of the analysis of 107 isolates on microarrays in terms of nucleotide profiles, MLST-predicted genogroups and sequence types obtained by Sanger sequencing. All MLSTs obtained by Sanger sequencing were shown to be part of a genogroup predicted by the mini-MLST scheme using microarrays. The sample of 107 isolates contained a total of 17 MLST genogroups, with two isolates categorized as ‘double strains’ (ST6726 or ST13758). The most common genogroups were G1594 (36 isolates), G1901 (19 isolates), and G1892 (16 isolates).

## 3. Discussion

Genotyping is an important and powerful tool for monitoring the causative agent of gonococcal infection, especially given the increasing problem of antimicrobial resistance of *N. gonorrhoeae*. MLST is a widely used method for genotyping *N. gonorrhoeae*, but its routine analysis of clinical isolates is associated with high labor costs. Mini-multilocus sequence typing is an alternative and less labor-intensive genotyping scheme based on the analysis of only informative SNPs in gene loci that are used for MLST genotyping. It is less labor-intensive and has sufficient predictive power. Mini-MLST has been successfully applied for rapid analysis of large samples of isolates, tracking epidemiologic events, and monitoring antibiotic-resistant strains.

This scheme has been used to analyze several bacteria including *P. aeruginosa*, *K. pneumoniae*, *S. aureus*, *E. faecium*, *E. coli*, etc. [25,31,32]. The mini-MLST scheme for *N. gonorrhoeae* requires further development as it has not been tested on a global population or large sample. Therefore, we propose an extension of the mini-MLST molecular typing scheme for *N. gonorrhoeae*, which takes into account the global epidemiological situation. This extension could facilitate the genotyping of gonococci in the future.

Whiley et al. and Trembizki et al. previously analyzed local samples of *N. gonorrhoeae* isolates using 14 informative polymorphisms [26,27]. The samples were examined through RT-PCR and the MassARRAY iPLEX platform [26,27]. Expanding the sample size of the current study to the global population of 21,402 *N. gonorrhoeae* isolates revealed 18 informative polymorphisms, which improved the theoretical accuracy of the mini-MLST prediction method. The accuracy of predicting the MLST type is 90%. Importantly, even when the MLST type is not accurately determined, the predicted type is phylogenetically similar in more than 98% of the isolates with the dominant MLST type and thus is potentially linked to the same resistance and pathogenicity determinants.

Additionally, only spontaneous substitutions occur in the analyzed loci, with their proportion not exceeding 1%. This may be due to natural heterogeneity or sequencing errors. Therefore, by analyzing the highly conserved MLST loci in the global population, we were able to expand the mini-MLST molecular typing scheme for *N. gonorrhoeae*, making it more suitable for mass screening. In view of the above, the introduction of a two-step genotyping system seems justified. The first step would be screening with genotyping methods such as NG-MAST or MLST/mini-MLST, while the second step would be genotyping selected isolates with cgMLST.

A mini-MLST profile of 18 informative nucleotides was obtained for each sequence type present in the global *N. gonorrhoeae* population. A new system for grouping individual sequence types into MLST genogroups was proposed based on the mini-MLST nucleotide profiles. On average, 90% of the genogroup was represented by only one sequence type, while the proportion of other sequence types within a single genogroup was negligible. The phylogenetic analysis of the global population of *N. gonorrhoeae*, taking into account MLST genogroups, indicates that most sequence types within a genogroup are closely related. Typically, each genogroup occupies a significant portion of the corresponding clade on the tree. Thus, MLST genogroups are a stable characteristic of the global population of *N. gonorrhoeae* and can be used for population genetic studies. It is important to note that the proposed system of sequence type grouping can be used with both the classic MLST scheme, which requires the full sequence of seven MLST loci, and the mini-MLST scheme, which only requires the determination of 18 informative polymorphisms.

An analysis of the most common *N. gonorrhoeae* genogroups in the world indicates that our proposed MLST grouping system brings together isolates with similar susceptibility to antimicrobial drugs. The distribution of MIC to recommended antimicrobials in different MLST genogroups is consistent with the literature data [14,33]. However, in our study, we analyzed the MLST genogroups obtained instead of individual sequence types. This simplified the holistic analysis of the global population of gonococcus.

The results of the MIC value comparison for ceftriaxone, cefixime and azithromycin for the major MLST genogroups (G1901, G7363, G1594, G9363 and G1588) are statistically significantly different (*p*-value < 0.001). Isolates from the G1901 genogroup were mostly associated with decreased susceptibility to third-generation cephalosporins (ceftriaxone and cefixime) due to the presence of mosaic *penA* alleles, particularly type “XXXIV”. Isolates from the G9363 genogroup are associated with resistance to azithromycin due to the mosaic *mtrCDE* operon and mutations in 23S rRNA.

To analyze *N. gonorrhoeae* isolates using the mini-MLST scheme, we have developed online and offline algorithms, available at https://minimlst.su/, accessed on 15 December 2023 and https://github.com/AnatoliyLarkin/mini-MLST, accessed on 1 April 2024, respectively. These algorithms can be effectively used with molecular methods that have a single-nucleotide resolution. It should be noted that the correctness of the developed algorithm depends on the accuracy of the pubMLST database. Some of these ST sequences resembled those of *N. meningitidis*, e.g., MLST: 12529, 12239, 13961, 15271, 15272, 15616, and 16263. This phenomenon could be attributed to either database errors or the results of horizontal gene transfer.

This work proposes a new method for analyzing clinical isolates of *N. gonorrhoeae* using the mini-MLST scheme based on DNA microarrays. The microarray allows for the simultaneous determination of 18 informative polymorphisms, significantly reducing the labor cost of the analysis in comparison with Sanger sequencing. The proposed method offers several advantages. It is rapid, inexpensive, and simple, both in terms of the result and its interpretation. The turnaround time of microarray analysis is less than eight hours, which is an advantage over the Sanger sequencing-based MLST scheme. With regard to the cost of analysis, the microarray is significantly less expensive than NGS and is comparable to PCR, with a per-sample cost of less than 10 USD. The key limitations of microarray-based assays are the incomplete automation of all steps of the procedure and the lack of standardization, which can be important for routine genetic testing in clinical laboratory settings. Nevertheless, some hydrogel microarray-based assays have been approved for in vitro clinical diagnostics by the Russian regulatory agency [34]. The developed method is limited by the lack of completeness of genetic information on both resistance determinants and pathogenicity factors. *De novo* resistance determinants arising within a characterized mini-MLST type are not always detectable.

The method was validated on clinical isolates from Russia and showed high predictive accuracy up to the MLST genogroup. The analysis of clinical isolates of *N. gonorrhoeae* using the developed DNA microarray and mini-MLST scheme demonstrated high efficiency for gonococcal genotyping. Overall, the MLST prediction method, which uses 18 informative polymorphisms, is a simple alternative to existing genotyping schemes.

## 4. Materials and Methods

### 4.1. Examination of N. gonorrhoeae Housekeeping Genes and Determination of Mini-MLST Profiles

The alleles of seven housekeeping genes of *N. gonorrhoeae* were analyzed using the PubMLST database (accessed on 15 December 2023). A total of 21,402 *N. gonorrhoeae* isolates with known MLSTs (1442 STs) were examined. A unique nucleotide sequence was constructed for each ST by concatenating the sequences of the *abcZ*, *adk*, *aroE*, *fumC*, *gdh*, *pdhC*, and *pgm* gene loci. The total length of the sequence is approximately 3286 bp. The resulting 1442 sequences were aligned using ClustalW v.2.1 (http://www.clustal.org/download/2.1/, accessed on 15 December 2023). The alignment was analyzed in Unipro UGENE v.44 [35] by calculating the SNP density using the nucleotide frequency module. The SNP density (%) was calculated as the ratio of the major and minor nucleotides in a single alignment position. A SNP density of 0% indicated the presence of only the major nucleotide in a specific position, while 50% indicated an equal ratio of major and minor nucleotides in that position.

SNP density values were obtained for all 3286 positions in the multiple alignment. Based on these values, 18 positions with densities greater than 5% were selected as informative SNPs. For each of the 1442 STs, we obtained a mini-MLST profile consisting of eighteen informative SNPs. The 1442 mini-MLST nucleotide profiles, each 18 bp long, were named after the corresponding MLST genotype number.

### 4.2. Definition of the MLST Genogroups and Generating a Voronoi Diagram

If the mini-MLST profile was identical, more than two MLSTs were merged into a genogroup. Genogroups were assigned the number of the ST with the highest frequency of occurrence worldwide among isolates. For instance, G1901 genogroup was formed by combining ST1901, ST6814, ST9365 and other sequence types due to their identical mini-MLST profile, and ST1901 is the most common sequence type in the *N. gonorrhoeae* global population. Mini-MLST profiles identified in only two distinct MLST genotypes were labeled as ‘double strains’ and were not merged into genogroups. Sequence types with a unique mini-MLST profile were classified as ‘single strains’. To analyze the genogroups and frequencies of STs in the *N. gonorrhoeae* global population, we constructed a Voronoi diagram for all 1442 MLSTs using the Voronoi Treemap v.1.1.2 package for R (with 100 iterations). The major genogroups in the world population, consisting of 250 or more isolates, were identified.

### 4.3. Constructing a Phylogenetic Tree (Phylogram)

Maximum likelihood phylogeny was constructed using RaxML v.8.2.4 (https://usegalaxy.eu/, accessed 15 December 2023) with 1000 rapid bootstrap inferences and the previously obtained multiple alignment of 1442 concatenated sequences of *abcZ*, *adk*, *aroE*, *fumC*, *gdh*, *pdhC*, and *pgm* loci. The phylogenetic tree was visualized in FigTree v.1.4.4 (http://tree.bio.ed.ac.uk/, accessed on 15 December 2023).

### 4.4. Development of the Mini-MLST Typing Tool for N. gonorrhoeae

The typing algorithm for *N. gonorrhoeae*, which is based on the mini-MLST scheme, was coded in the JavaScript programming language using the Eclipse IDE development environment (https://www.eclipse.org/ide/, accessed on 15 December 2023). The database of acquired mini-MLST profiles was integrated with the procedure of grouping STs into genogroups and assigning them to ‘double’ and ‘single’ strains, along with the Voronoi diagram. The frontend is coded in the HTML programming language and is publicly available at the following link (https://minimlst.su/, accessed on 15 December 2023). The algorithm for offline typing is written in the Python programming language and is available on the GitHub repository page (https://github.com/AnatoliyLarkin/mini-MLST, accessed on 1 April 2024 and Supplementary File S2).

### 4.5. Analysis of Antimicrobial Susceptibility and Construction of Box Plots Diagrams

Box plot diagrams were constructed in the software package ggplot2 for R (https://www.rdocumentation.org/packages/ggplot2/versions/3.5.0/, accessed on 15 December 2023). Five MLST genogroups with the largest number of sequence types were selected for analysis: G1901 (59 STs), G7363 (44 STs), G1594 (29 STs), G9363 (24 STs), and G1588 (23 STs). The sample consisted of all isolates from the PubMLST database belonging to selected genogroups with known minimum inhibitory concentrations (MICs) to ceftriaxone (4873 STs), cefixime (4569 STs), and azithromycin (4636 STs), as shown in Table S2 (Supplementary Materials). For each MLST genogroup, we determined the median and mean MIC to antimicrobials, as well as the lower and upper quartiles, interquartile range, and outliers. To compare MIC between the mentioned genogroups, we employed Dunn’s test for multiple comparisons using rank sums, where the calculation of the Benjamini-Hochberg correction for the adjusted *p*-value was calculated using the dunn.test software package version 1.3.6 for R (https://cran.r-project.org/web/packages/dunn.test/index.html, accessed on 7 May 2024).

### 4.6. Microarray-Based Assay for the Analysis of N. gonorrhoeae Isolates Using the Mini-MLST Scheme

**Figure 6:**
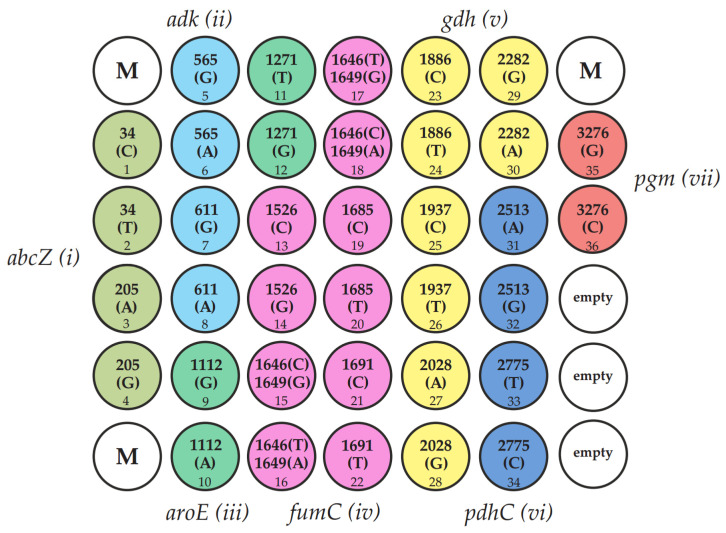
DNA hydrogel microarray for SNPs identification used in the mini-MLST scheme of *N. gonorrhoeae*. SNPs analyzed are indicated in circles representing elements of the microarray. The microarray included 36 hydrogel elements containing immobilized oligonucleotide probes, three marker elements (M) for imaging and processing, and three empty hydrogel elements for signal normalization. The microarray elements were divided into seven sets of elements labeled with different colors that correspond to the genes to be analyzed. The microarray provides detection of informative polymorphisms in the *abcZ* gene-set i (positions 34, 205), *adk* gene-set ii (positions 565, 611), and *aroE* gene-set iii (positions 1112, 1271), *fumC* gene-set iv (positions 1526, 1646, 1649, 1685, 1691), *gdh* gene-set v (positions 1886, 1937, 2028, 2282), *pdhC* gene-set vi (positions 2513, 2775), and *pgm* gene-set vii (position 3276).

The identification of 18 informative SNPs was carried out using a molecular technique that utilizes hydrogel-based oligonucleotide microarray technology developed at the Engelhardt Institute of Molecular Biology, Russian Academy of Sciences [34]. The microarray consisted of a plastic substrate with attached hydrogel elements (droplets) containing immobilized oligonucleotides. The process of designing and synthesizing oligonucleotides for immobilization, along with the manufacturing of microarrays using a copolymerization immobilization method, follows the previously described protocol [36]. Supplementary File S1 provides the sequences of the immobilized probes. Figure 6 displays the layout of the microarray that contains oligonucleotides for identifying 18 informative SNPs.

The assay protocol involved multiplex PCR to generate predominantly single-stranded, fluorescence-labeled DNA fragments, followed by hybridization of the DNA fragments to a microarray and acquisition and analysis of fluorescence images. The primer sequences used for the multiplex PCR and the procedures for performing the PCR and microarray hybridization are outlined in Supplementary File S1. A proprietary microarray scanner equipped with original software (Biochip-IMB, Ltd., Moscow, Russia) was used to acquire fluorescence images and measure signal intensities. In each set of microarray elements corresponding to an individual informative SNP, the maximum signal matching the ideal hybridization duplex was registered. Based on the results of genomic DNA analysis of the *N. gonorrhoeae* isolate using a microarray, a sequence of 18 informative oligonucleotide polymorphisms was obtained and used to compile a mini-MLST profile. The obtained profile was compared to the MLST-specific profiles presented in Table S1 of the Supplementary Materials, utilizing the principle of maximum concordance to establish its association with the MLST genogroup.

## 5. Conclusions

In recent years, several methods for *N. gonorrhoeae* genotyping have emerged. The main trend in their development is to increase the number of analyzed loci and information obtained. Examples of these methods include 16S rRNA typing schemes, NG-MAST, MLST, and cgMLST. The benefits of utilizing more powerful methods are apparent. However, there is a higher cost and a lack of a methodological approach that prevents prompt classification of obtained genotypes and comparison with well-characterized genotypes in the population. Our proposed holistic approach to *N. gonorrhoeae* population analysis based on the mini-MLST scheme is simple and fast. It solves the stated methodological problems and is, to some extent, a development of anti-reductionist ideas [37].

## Data Availability

An online tool for the analysis of *N. gonorrhoeae* isolates is available in the public domain (https://minimlst.su/, accessed on 1 April 2024). An offline tool for the analysis of *N. gonorrhoeae* isolates is available on the GitHub repository page (https://github.com/AnatoliyLarkin/mini-MLST, accessed on 1 April 2024).

## Supplementary Materials

The following supporting information can be downloaded at: https://www.mdpi.com/article/10.3390/ijms25115781/s1.
