# Supplementary material for: Mini-Multilocus Sequence Typing Scheme for the Global Population of Neisseria gonorrhoeae
Source: Int J Mol Sci. 2024 May 26;25(11):5781. doi: 10.3390/ijms25115781 (PMC11171571; doi:10.3390/ijms25115781)
Supplement: Supplementary file 1 [file ijms-25-05781-s001.zip › Supplementary File S1.pdf]

Supplementary File S1. Identifying informative polymorphisms in *Neisseria gonorrhoeae* MLST-related genes using a microarray-based assay

Microarray layout

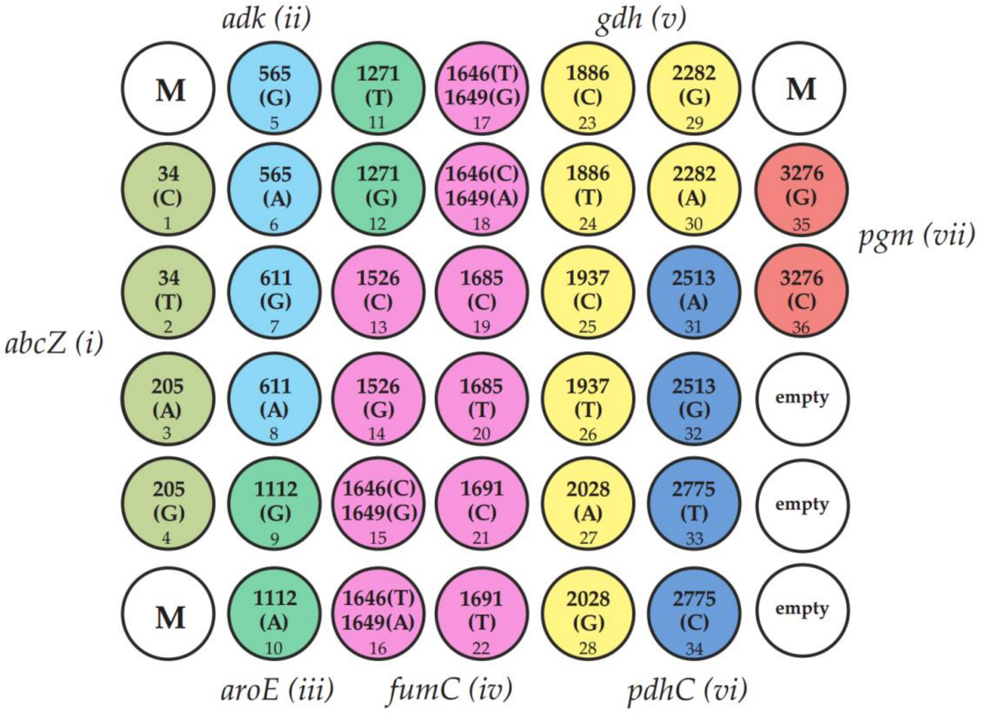

List of oligonucleotides immobilized on the microarray elements

	Gene	Set	Position of analyzed nucleotide	Probe sequence, 5'-3'	Length, b.p.
1	*abcZ*	i	34(C)	GGCGAAATTCGCGATTTATT	20
2	*abcZ*		34(T)	GGCGAAATTTGCGATTTATT	20
3	*abcZ*		205(A)	GCCGGAAAACAAAAAAATCG	20
4	*abcZ*		205(G)	GCCGGAAAACGAAAAAATCG	20
5	*adk*	ii	565(G)	GTACATTGGCGCAAGCCGAA	20
6	*adk*		565(A)	GTACATTGGCACAAGCCGAA	20
7	*adk*		611(G)	GACGCGGTCGTTGAAATCGA	20
8	*adk*		611(A)	GACGCGGTCATTGAAATCGA	20
9	*aroE*	iii	1112(G)	GGCGGATGTGAACGGCGGTT	20
10	*aroE*		1112(A)	GGCGGATGTAAACGGCGGTT	20
11	*aroE*		1271(T)	CGACAATCGGGTGCGAAAAAA	21
12	*aroE*		1271(G)	CGACAATCGGGGGCGAAAAAA	21
13	*fumC*	iv	1526(C)	AATCAAAATCCCCGAAAACG	20
14	*fumC*		1526(G)	AATCAAAATGCCCGAAAACG	20
15	*fumC*		1646(GC)	TATGGCGGGCGCGTCGGGCAA	21
16	*fumC*		1646(TA)	TATGGCGGGTGCATCGGGCAA	21
17	*fumC*		1649(TG)	TATGGCGGGTGCGTCGGGCAA	21

18	*fumC*		1649(CA)	TATGGCGGGCGCATCGGGCAA	21
19	*fumC*		1685(C)	TATGCCCGTCATCGCCTACA	20
20	*fumC*		1685(T)	TATGCCCGTTATCGCCTACA	20
21	*fumC*		1691(C)	CCGTTATCGCCTACAACCTC	20
22	*fumC*		1691(T)	CCGTTATCGCTTACAACCTC	20
23	*gdh*	v	1886(C)	TAAATACATCGAAAGCGTGC	20
24	*gdh*		1886(T)	TAAATACATTGAAAGCGTGC	20
25	*gdh*		1937(C)	AGAGCGCGGCGAGTTTTACG	20
26	*gdh*		1937(T)	AGAGCGCGGTGAGTTTTACG	20
27	*gdh*		2028(A)	GCCCCCGCCAGCTTGGATG	19
28	*gdh*		2028(G)	GCCCCCGCCGGCTTGGATG	19
29	*gdh*		2282(G)	ACGCATGGCGGGCAAAGTGG	20
30	*gdh*		2282(A)	ACGCATGGCAGGCAAAGTGG	20
31	*pdhC*	vi	2513(A)	CGTGGAAGCAGACATCTGGT	20
32	*pdhC*		2513(G)	CGTGGAAGCGGACATCTGGT	20
33	*pdhC*		2775(T)	CTGCGCCGCTTCTTTGAAGT	20
34	*pdhC*		2775(C)	CTGCGCCGCCTCTTTGAAGT	20
35	*pgm*	vii	3276(G)	GCCAAAGCCGAATTTGAAGG	20
36	*pgm*		3276(C)	GCCAAAGCCCAATTTGAAGG	20
empty	*Empty gel*	-	-	-	-
M	*Marker elements*	-	-	-	-

Multiplex PCR

Two rounds of multiplex PCR were performed to amplify the *abcZ*, *adk*, *aroE*, *fumC*, *gdh*, *pdhC*, and *pgm* loci. The reaction mixture consisted of DNA polymerase (SibEnzyme, Russia), dNTPs (Evrogen, Russia), fluorescently labeled Cy5-dUTP (Lumiprobe, Russia), and the corresponding forward and reverse primers (refer to the Table below). The template used for the first round of amplification was *N. gonorrhoeae* genomic DNA (1 µl per mixture). PCR was performed using an S1000 thermal cycler (Bio-Rad, USA) with the following conditions: initial denaturation at 95°C for 5 minutes, followed by 15 cycles of denaturation at 95°C for 30 seconds, annealing at 65°C for 30 seconds, and extension at 72°C for 30 seconds.

For the second round of amplification, PCR fragments obtained during the first amplification round (1 µl each) were used as a template. PCR was performed with the following conditions: initial denaturation at 95°C for 5 minutes, followed by 30 cycles of denaturation at 95°C for 30 seconds, annealing at 65°C for 30 seconds, and extension at 72°C for 30 seconds.

The mixtures were supplemented with forward and reverse primers for the second round of amplification (Table below). To obtain predominantly single-stranded DNA fragments, excess reverse primers were added. The mixtures of PCR products, which contained mainly single-stranded fluorescently labeled fragments after the second round of amplification, were combined and utilized for hybridization on microarrays.

Primers for the first amplification round

	**Direction**	**Gene**	**Sequence, 5'-3'**	**Concentration in the reaction mixture (mM)**
1	for	*abcZ*	AATCGTTTATGTACCGCAGG	2
2	rev	*abcZ*	GAGAACGAGCCGGGATAGGA	2
3	for	*adk*	AGGCAGGCACGCCCTTGG	2
4	rev	*adk*	CAATACTTCGGCTTTCACGG	2
5	for	*aroE*	GCGGTCAATACGCTGGTG	2
6	rev	*aroE*	ATGATGTTGCCGTACACATA	2
7	for	*fumC*	TCCGGCTTGCCGTTTGTCAG	2
8	rev	*fumC*	TTGTAGGCGGTTTTGGCGAC	2
9	for	*gdh*	CCTTGGCAAAGAAAGCCTGC	2
10	rev	*gdh*	GCGCACGGATTCATTCGG	2
11	for	*pdhC*	TCTACTACATCACCCTGATG	2
12	rev	*pdhC*	ATCGGCTTTGATGCCGTATTT	2
13	for	*pgm*	CGGCGATGCCGACCGCTTGG	2
14	rev	*pgm*	GGTGATGATTTCGGTTGCGCC	2

Primers for the second amplification round

	**Direction**	**Gene**	**Sequence, 5'-3'**	**Concentration in the reaction mixture (mM)**
1	for	*abcZ*	AATCGTTTATGTACCGCAGG	1
2	rev	*abcZ*	CCAGTAACAATACATCGGGC	15
3	for	*adk*	GGTCAAAGAACGCATCGCG	1
4	rev	*adk*	GTAGGTAACGTGGTAAGTACGG	10
5	for	*aroE*	ATTGCCAACCGTACCCG	1
6	rev	*aroE*	AGAGGGCGTAGGAAGCC	10
7	for	*fumC*	CCTGAACAAAATCGCCAACG	1
8	rev	*fumC*	AGTTTTCGTTGAAGCTGTTGC	10
9	for	*gdh*	CCTTGGCAAAGAAAGCCTGC	1
10	rev	*gdh*	GACAGATTCGATGGTCAGCG	10
11	for	*pdhC*	TATGCCGACCGTATCCGC	1
12	rev	*pdhC*	ATCGGCTTTGATGCCGTATTT	10
13	for	*pgm*	AAGTGTTGGACAACCTGCC	1
14	rev	*pgm*	GGAAGCACGCATCAGACC	20

Microarray hybridization

The Multiplex PCR product mixture (20 μL) was added to 10 μL of hybridization buffer (0.3 M HEPES (pH 7.5), 3.0 M guanidine thiocyanate, 30 mM EDTA). The microarray hybridization chambers were filled with the mixtures (30 μL), and the microarrays were incubated at 37°C for 6-12 hours. After incubation, the hybridization chambers were removed, and the microarray surfaces were washed three times with distilled water and air-dried.
